# Employment predictors of exit from work among workers with disabilities: A survival analysis from the household income labour dynamics in Australia survey

**DOI:** 10.1371/journal.pone.0208334

**Published:** 2018-12-07

**Authors:** Allison Milner, Yamna Taouk, George Disney, Zoe Aitken, Jerome Rachele, Anne Kavanagh

**Affiliations:** 1 Centre for Health Equity, Melbourne School Population and Global Health, The University of Melbourne, Victoria, Australia; 2 Centre for Health Policy, Melbourne School Population and Global Health, The University of Melbourne, Victoria, Australia; Universita degli Studi di Perugia, ITALY

## Abstract

**Objectives:**

Across high-income countries, unemployment rates among workers with disabilities are disproportionately high. The aim of this study was to identify characteristics of employment associated with dropping out of work and assess whether these were different for workers with versus without disabilities.

**Methods:**

Using a longitudinal panel study of working Australians (2001 to 2015), the current study estimated Kaplan–Meier curves and Cox proportional hazard regression models to identify predictors of leaving employment, including psychosocial job quality, employment arrangement, and occupational skill level. Effect modification by disability status of the relationship between employment-related factors and exit from the labour market were assessed by including interaction terms and assessing model fit with a likelihood ratio test. Models were adjusted for a range of socio-demographic and health related factors.

**Results:**

Compared to those without disability, those with disability had a greater risk of leaving employment (HR 1.26, 95% CI 1.18 to 1.35, p<0.001). Other predictors of exit from work included low-skilled occupation (HR 1.18, 95% CI 1.07 to 1.29, p = 0.001), being in a job with low psychosocial job quality (HR 1.11, 95% CI 1.03 to 1.19, p = 0.007), and casual, labour hire or fixed-term contract employment (HR 1.58, 95% CI 1.48 to 1.69, p<0.001). There was no effect modification by disability status.

**Conclusions:**

More research is needed to understand the experiences of workers with disabilities who stay in and leave employment.

## Introduction

Across many high-income countries, people with disabilities are less likely to be employed than people without disabilities [1]. In Australia, the rate of employment among people with disabilities is 48.1% compared to 78.8% among people without disabilities [2]. From a public health perspective, it is critical to understand the range of factors that inhibit people with disabilities from engaging in employment. There is some evidence that, at a population level, being employed increases mental health [3]. At the same time, there may also be some health selection effects, in that the healthiest people are likely to be employed [4, 5]. Regardless, employment appears to be particularly important to the wellbeing of people with disabilities [6, 7]. However, previous research has also shown that people with disabilities are disproportionately employed in jobs with lower control and greater levels of job insecurity than their non-disabled peers [8]. This is problematic considering the growing body of evidence regarding the association between poor psychosocial job quality and stress-related disorders [9].

Past research has tended to focus on the factors that inhibit people with disabilities from getting into work [10–13]. There has been less focus on the factors predicting exit from paid work among those with disabilities. There is however, substantial research on predictors on return to the labour market for specific conditions, such as injury [14] or trauma [15]. For example, Kendrink et al [14] found that, following an injury, women, those with more severe injuries, those who were injured at work, and those living in socioeconomically deprived areas were less likely to return to the labour force. Similar factors (e.g., illiteracy, drug abuse, hospitalization history in the intensive care unit, low socioeconomic status, non-insurance coverage, multiple injuries as well as severe disability) have been identified as important predictors of return to work following trauma [15]. However, these studies are focused on specific injuries or trauma rather than disabilities in general, which covers a wider range of long-term health conditions and impairments restricting everyday activities. Furthermore, most previous research has largely used health or hospital-based samples rather than a representative sample of people with disabilities [14, 16, 17]. It is unknown whether the results of these studies apply to a wider range of persons with disabilities.

As mentioned above, previous research has tended to focus on specific conditions rather than disability as a broader construct. In disability research, there has been a movement away from the medical model, where specific health conditions or illnesses are nominated as being disabilities. The reason for this is that this model does not recognise severity of disability (e.g., level of impairment), activities and participation (e.g. dressing or participation in society), or environmental factors (e.g. barriers or facilitators to participation) (1, 2), all of which impact the extent to which a person is able to stay in employment. The International Classification of Disability, Functioning and Health (ICF) [18] attempts to conceptualise this broader understanding of disability using a biopsychosocial approach [19]. The measurement of disabilities in the current study uses a definition that captures the extent to which disability restricts everyday activities that lasts (or could last) for six months or more.

The aim of this paper is to examine the employment-related predictors of exit from work among workers with disabilities and assess whether these differ from those workers without disabilities. There has been some work in economics documenting the difference in employment participation and arrangements (e.g., moving between full time and part time work) among people once they acquire a disability [20–24], noting that these have used different research designs than we proposed and (importantly) have not been able to examine important characteristics of the working environment, such as psychosocial job quality, as predictors of exit from work. Understanding what contributes to loss of work among people with disabilities is important considering the evidence that: 1) loss of employment is associated with substantial declines in the mental health of people with disabilities [6], and; 2) people with disabilities are more exposed to poor psychosocial job quality, unemployment and NILF [6, 8]. Using 15 waves of a general working age population cohort, the study will use a survival analysis approach and assess for effect modification between workers with and without disabilities. We hypothesise that employment predictors of exit from work will differ between those persons who do and do not have a disability.

## Materials and methods

### Data source

The Household, Income and Labour Dynamics in Australia (HILDA) survey is a longitudinal, nationally representative study of Australian households established in 2001. It collects detailed information annually from over 13,000 individuals within over 7,000 households [25]. A top-up sample of 2,000 responding households, totalling 4000 responding people, was added in 2011 to allow better representation of the Australian population using the same methodology as the original sample [26]. The response rates for the HILDA survey are above 90% for respondents who have continued in the survey and above 70% for new respondents being invited into the study [25]. The main variables examined in this study were available in all annual waves of HILDA (2001 to 2015). More information on the HILDA survey is available in S1 File.

### Outcome variable

We considered employment (yes or no) as our primary outcome. A person was considered to have “failed” if they dropped out of work, either because they became unemployed (e.g., where they were still actively looking for work) or were “not in the labour force” (i.e., NILF), where a person was not actively searching for work. As mentioned below, we conducted a sensitivity analysis to examine differences in outcomes depending on whether a person was unemployed or NILF. A key inclusion criterion was that a person had been in employment for at least one wave before they could be considered at-risk of experiencing exit. A person was excluded from the study once exit occurred.

### Exposure variable

Disability was determined from the following survey question *“…do you have any long-term health condition*, *impairment or disability that restricts you in your everyday activities*, *and has lasted or is likely to last*, *for six months or more*?*”* Specific examples of long-term conditions were shown, such as limited use of fingers or arms, or problems with eyesight that could not be corrected with glasses or contact lenses. These questions were asked at every wave. Data was available from 2001 to 2015. Information on impairments (available from 2003 onwards) suggests that most people reported a physical impairment (6.12% of the observations in HILDA) followed by unspecified impairments (5.88% of the observations in HILDA), sensory (2.51% of the observations in HILDA) and psychosocial problems (1.64% of the observations in HILDA).

We constructed a time-invariant measure of disability that identified if a person reported disability in any waves versus no waves (non-disability). Within the disability group, we conducted a sensitivity analysis to assess possible differences between the proportion of the time a person reported a disability (between 1 to 24% of the time they were in the cohort, 25% to 49% of their time in the cohort, 50% to 74% of their time in the cohort, and 75% to 100% of their time in the cohort).

### Predictors of exit

#### Employment predictors of exit

The following time-varying employment-related predictors of dropping out of work included: employment arrangement (permanent, casual or labour hire or fixed-term contract, self-employed), occupational skill level (low [sales, machinery workers, and labourers], medium [technical and trade workers, community and personal service workers, and clerical and admin workers], and high [managers and professionals] according to the Australian and New Zealand Standard Classification of Occupations occupational groupings) [27]. We also hypothesised that psychosocial job quality may be a predictor of dropping of work, and measured this using a multidimensional measure of four psychosocial job stressors (job control, demands and complexity, job insecurity, and unfair pay). This was coded as ranging from optimal jobs (no report of psychosocial job stressors) to one or more psychosocial adversities (lowest quality jobs). Full details of the construction and validation of the psychosocial job quality measure are presented elsewhere [28–30].

#### Confounders

Time-varying confounders included general health and wellbeing (obtained from the 36-Item Short Form Survey (SF-36) Mental and Physical Health Component summary scales (MCS and PCS) [31]), age (measured continuously), and household income, using a categorical variable relating to quintiles of the population distribution of disposable household income. Household disposable income was calculated by summing the income components for the previous financial year for all adults in the household (gross regular income minus taxes) and equivalised using the modified OECD scale [32]. Missing values were imputed using nearest neighbour imputation (20% imputed values for observations in the sample) [33, 34]. For each wave of data, nominal household income values reported by sample members were converted to quintiles of the Australian population distribution using percentile statistics for the corresponding financial year published by the ABS from the biennial Survey of Income and Housing [35]. We also adjusted for area of residence (metropolitan, inner regional, and outer regional or remote coded using the 2011 Australian Statistical Geography Standard from the Australian Bureau of Statistics) [36]. Time-invariant variables included: education (less than year 12 (high school), year 12, diploma or certificate, bachelor degree), gender (male or female), and country of birth (Australia, other English speaking country, other non-English speaking country). It is worth note that we made a distinction between the measurement of disability (measured as a time-invariant factor) and general health and wellbeing (time-varying). People with disability may have differing perceptions of their own health from wave one wave to another, which supports the measurement of the SF-36 MCS and PCS as time-varying confounder of the relationship between dropping out of work and employment in different types of jobs and working arrangements.

### Analytic approach

We constructed a survival analysis dataset with multiple observations per person. To be considered eligible for inclusion in the analysis a person had to be employed for one wave of employment before becoming observed as “at risk” of dropping out of employment. We considered time of origin as the first period a person was defined to be at risk (e.g., they were employed for at least once wave). People were censored if they had missing data on the outcome (e.g., once a person had missing data on employment), or did not observe the outcome at the end of their last contributed wave to HILDA. People “failed” once they reported being unemployed or “not in the labour force”. The study population was restricted to people between the ages of 17 and 64 years.

We estimated and plotted survival curves to assess differences in the outcome, over time, by disability status (two levels (yes/no)). We then used a log-rank test of equality of the survivorship functions to examine whether there were differences in exit from employment by disability.

Semi-parametric Cox regression models were used to examine time-varying predictors of exit from paid work those who did and did not experience any disability. A sensitivity test was performed to assess for possible differences depending on the proportion of time a person reported disability. We controlled for all identified time-varying and time-invariant confounders as discussed above. To determine whether disability was an effect modifier of employment-related factors and exit from employment, we assessed interaction terms of disability and employment variables. We checked if the interactions improved the fit of our model to the data using the likelihood ratio test (comparing a model with multiplicative effects of disability and employment to a model with main effects only).

To deal with the potential selection bias associated with who was left remaining in the sample at follow up (defined as not experiencing exit from employment), we derived weights representing the probability of remaining uncensored up to time *t* using the approach described in Fewell et al., [37]. We estimated the denominator using a logistic regression approach. The outcome was defined as remaining uncensored at end of study follow up. Specifically, participants whom remained in the sample were coded ‘1’; and censored participants were coded ‘0’. The predictors measured included time-varying and time-invariant covariates. In addition, a variable describing the number of years in the study and a spline variable representing the 5^th^, 25^th^, 50^th^, 75^th,^ 95^th^ percentile of number of years in the study were included in the model. Finally, to control for each participant’s censoring history, we calculated the probability of complete censoring history to each year for each individual included in the study. The numerator was estimated in a similar way to the denominator, except that the initial logistic regression only included covariates measured at baseline. The stabilised weights were calculated by dividing the denominator by the numerator, and a mean value was then calculated for each individual included in the study. A further sensitivity analysis was conducted to assess differences based on whether an individual left employment due to NILF versus unemployment. This was calculated across the whole model, as well as for persons with and without disabilities. We also assessed whether removing the SF-36 MCS and PCS influenced the main results regarding disability, employment and exit from employment.

## Results

**Fig 1:**
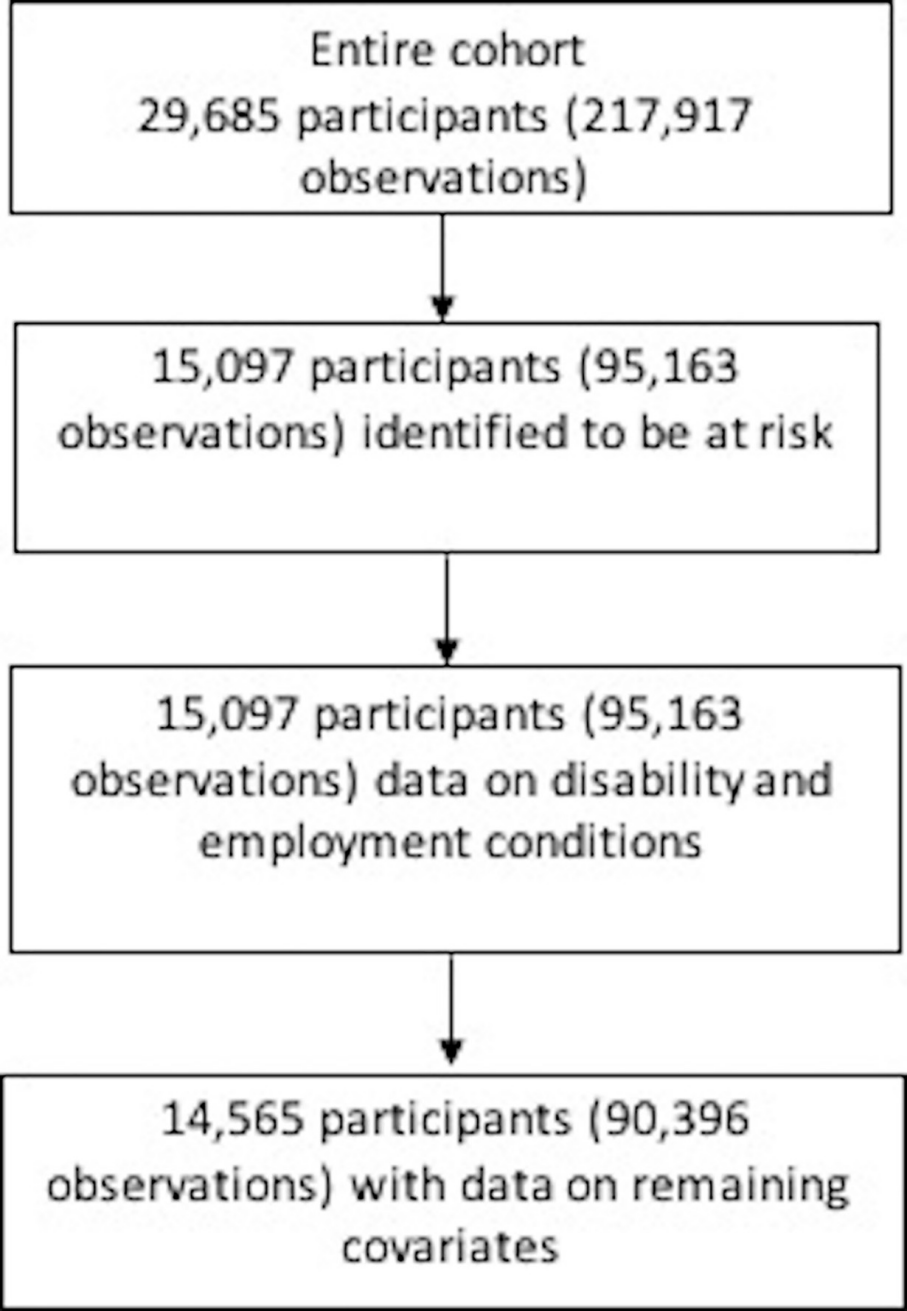
Sample selection.

The selection of participants is summarised in Fig 1. There were a total of 14,565 participants with complete data on disability and employment conditions; and no missing covariates, identified to be at risk, and 4,581 failures (e.g., persons leaving the labour market). Table 1 displays summary statistics of predictors of employment exit by disability status. Seventy-three percent of those with disability experienced at least one job stressor, and 71% of those with no disability reported at least one job stressor. About one-third of people were employed in a low-skilled job and 39% were employed in a medium-skilled job. Over half of participants (52%) were employed permanently. Fifteen percent of those with disability, and 11% of those with no disability were self-employed. A third of those with disability, and 36% of those with no disability were in casual of fixed-term employment.

**Table 1:** Sample characteristics at baseline.

		No disability (n = 8623) %		Disability (n = 5942) %	
Occupation	Low	2775	32.18	1870	31.47
	Medium	3304	38.32	2329	39.2
	High	2544	29.5	1743	29.33
Psychosocial job	High	2508	29.09	1580	26.59
quality	Low	6115	70.91	4362	73.41
Employment	Permanent	4486	52.02	3093	52.05
arrangement	Casual/LH/fixed-term	3149	36.52	1939	32.63
	Self-employed	988	11.46	910	15.31
SF-36 PCS	Mean, std. dev.	54.11	6.48	50.49	8.84
SF-36 MCS	Mean, std. dev.	49.5	9.13	47.94	10.3
Education	Postgraduate	799	9.27	514	8.65
	Bachelors degree	1481	17.17	789	13.28
	Dip or Cert.	2290	26.56	1848	31.1
	Year 12	1767	20.49	951	16
	Less than year 12	2286	26.51	1840	30.97
Age	17–24 years	3061	35.5	1201	20.21
	25–34 years	2220	25.75	1116	18.78
	35–44 years	1800	20.87	1450	24.4
	45–54 years	1126	13.06	1391	23.41
	55–64 years	416	4.82	784	13.19
Household	Couple no children	2166	25.12	1583	26.64
structure	Couple with children	4414	51.19	2785	46.87
	Lone	784	9.09	598	10.06
	Lone person	704	8.16	705	11.86
	Multi-person	555	6.44	271	4.56
Gender	Male	4308	49.96	2976	50.08
	Female	4315	50.04	2966	49.92
Country of birth	Australia	6848	79.42	4770	80.28
	Eng. speaking	753	8.73	626	10.54
	Other country	1022	11.85	546	9.19
Household	Lowest	514	5.96	471	7.93
income	Low-med	1263	14.65	961	16.17
	Medium	1903	22.07	1327	22.33
	Med-high	2352	27.28	1504	25.31
	Highest	2591	30.05	1679	28.26

Notes: Dip or Cert = Diploma or certificate; Lone = lone person with children; Eng speaking = Other English speaking; SF-36 MCS & PCS = 36-Item Short Form Survey Mental Health and Physical Health Component Summary Score. Household income (population quintiles) = Lowest (mean $293, std. dev. $481); Low-medium (mean $523, std. dev. $118); Medium (mean $ 714, std. dev. $ 152); Medium-high ($943, std. dev. 207); Highest (mean $1543, std. dev. $796)

**Fig 2:**
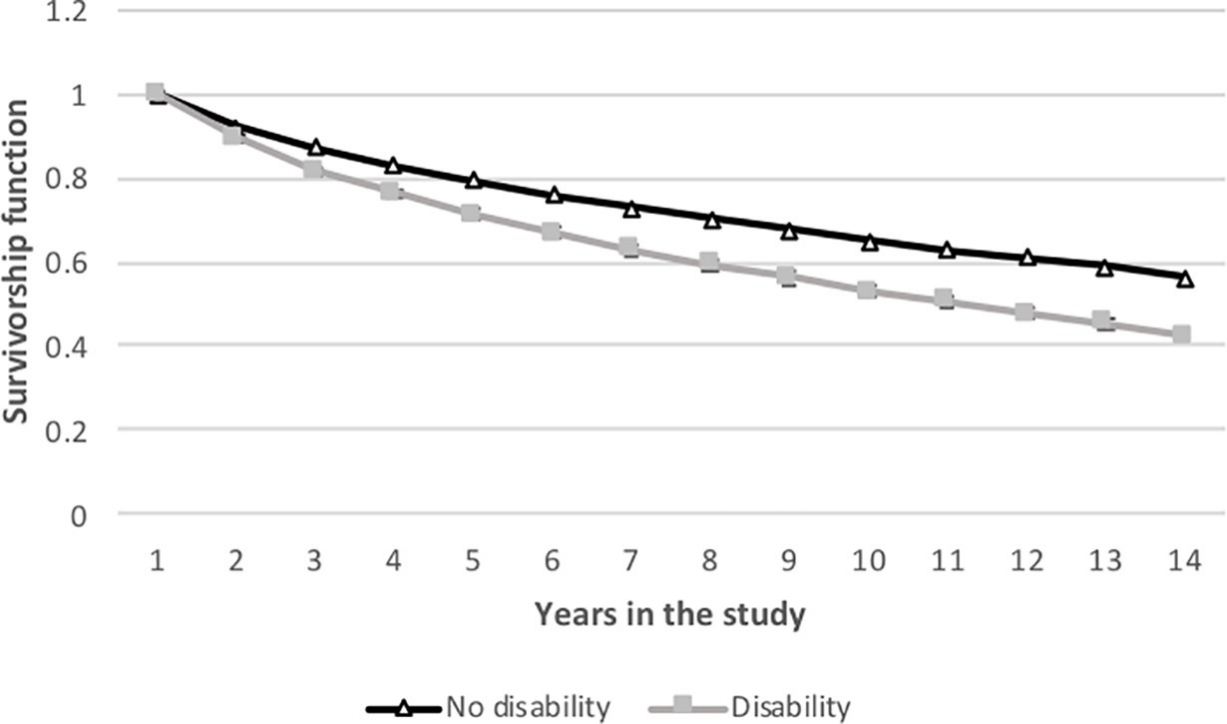
Time to leaving employment, by presence of disability, HILDA 2001 to 2015.

Survivor function graphs (Fig 2) identified notable differences in exit from employment between those who did and did not report disability. The probability of remaining in employment at the end of the study period was 0.42 (95% CI 0.41 to 0.44) for those with disability and 0.56 (95% CI 0.55 to 0.58) for those with no disability. The log rank test suggests significant differences in exit from employment by disability (e.g., survivor functions are not the same) (x^2^(1) = 176.79, p<0.001).

There was no significant interaction between disability and employment characteristics (results available in S1 Table). These results suggest that the same employment characteristics affected exit from the work for those people with and without disability (although the effect of psychosocial job quality just crossed to bounds into non-significance). The main effects of the analysis, predictors of labour market exit, can be seen in Table 2 (note: S2 Table shows results stratified by disability status). Compared to those without disability, those with disability had a greater risk of leaving employment (HR 1.26, 95% CI 1.18 to 1.35, p<0.001). Other predictors of exit from work included low-skilled occupation (HR 1.18, 95% CI 1.07 to 1.29, p = 0.001), being in a job with low psychosocial job quality (HR 1.11, 95% CI 1.03 to 1.19, p = 0.007), and casual, labour hire or fixed-term contract employment (HR 1.58, 95% CI 1.48 to 1.69, p<0.001). Our sensitivity analysis assessing possible differences depending on the proportion of time a person reported a disability were consistent with those reported above, but this lacked precision as the sample was divided into four groups (S3 Table). Our sensitivity analysis assessing differences in whether a person left employment for unemployment or NILF can be seen in S4 Table. These show that disability predicts exit into both states. There were some notable differences by age (older workers were less likely to exit into unemployment than younger workers but were more likely to exit onto NILF) and gender (women were more likely to exit into NILF, while men were more likely to exit into unemployment). Being in a low skill job and a poor-quality job predicted exit into unemployment more than NILF. Being casual/LH/fixed-term employed predicted exit in both states. Removing the SF-36 MCS and PCS from the main effects model did not change results (results available on request).

**Table 2:** Results of the Cox regression analysis, predictors of leaving employment, HILDA, 2001 to 2015 (persons = 14,565).

		HR	95% L CI	95% U CI	p value
Disability	No disability	1			
	Disability	1.26	1.18	1.35	<0.001
Occupation	High	1			
	Medium	1.07	0.98	1.17	0.124
	Low	1.18	1.07	1.29	0.001
Psychosocial	High	1			
job quality	Low	1.11	1.03	1.19	0.007
Employment	Permanent	1			
arrangement	Casual or fixed-term	1.58	1.48	1.69	<0.001
	Self-employed	0.96	0.86	1.07	0.428
SF-36	MCS	0.98	0.98	0.99	<0.001
	PCS	0.99	0.98	0.99	<0.001
Education	Postgraduate	1			
	Bachelors degree	0.95	0.83	1.09	0.488
	Diploma or certificate	0.95	0.83	1.08	0.453
	Year 12	0.99	0.86	1.14	0.902
	Less than year 12	1.04	0.91	1.19	0.548
Age	17–24 years	1			
	25–34 years	1.06	0.96	1.16	0.225
	35–44 years	0.73	0.66	0.81	<0.001
	45–54 years	0.63	0.56	0.7	<0.001
	55–64 years	1.36	1.22	1.52	<0.001
Household	Couple no children	1			
structure	Couple with children	1.36	1.25	1.48	<0.001
	Lone person with child	1.08	0.96	1.22	0.201
	Lone person	0.91	0.82	1.02	0.092
	Multi-person	1.15	0.98	1.34	0.079
Household	Lowest	1			
income	Low-med	0.68	0.62	0.75	<0.001
	Medium	0.53	0.48	0.58	<0.001
	Med-high	0.44	0.4	0.49	<0.001
	Highest	0.47	0.42	0.53	<0.001
Gender	Male	1			
	Female	1.42	1.33	1.52	<0.001
Country of	Australia	1			
birth	Other English speaking	1.04	0.93	1.15	0.498
	Other country	0.96	0.86	1.07	0.417
Remoteness	Major Cities	1			
	Inner Regional	1	0.92	1.08	0.901
	Outer Reg. and remote	1.01	0.92	1.11	0.861

Notes: Remoteness = remoteness of residence defined by the Australian Statistical Geography Standard; HR = Hazard Ratio; 95% Lower CI = Lower confidence interval at 95% significance; 95% Upper CI = Upper confidence interval at 95% significance; p value = significance at 95% significance.

## Discussion

The findings of this paper suggest that employed persons with disabilities exit from employment at a much greater rate than persons without disabilities. However, the employment factors predicting exit appear to be similar between those with and without disability when measured on a relative scale. Hence, we were not able to find support for the hypothesis that employment related predictors of exit differ between those with and without disability. The lack of difference in employment related predictors suggests that these factors are detrimental for all workers, as we discuss below. However, the effect of disability appears to operate over and above these employment predictors and also above the effect of health, as measured using the SF-36.

### Employment related predictors of exist from employment

These factors predicting exit included: being employed in a low skilled occupation, being employed casually or on a fixed term basis, and being employed in a low psychosocial quality job (noting that this just crossed the bounds of significance for those with disabilities). There is considerable research demonstrating higher unemployment rates among low-skilled and “casual” (a measure of employment precarity) employed workers across OECD countries [38, 39], which may explain the higher rates of employment exit in these groups. The explanations for this are complex, but likely are related to wider economic structural factors, including a poor labour market, overall levels of country debt and the absence of labour market programs [38, 39]. It might be the case that, regardless of the presence of disability, low skilled workers are more likely to be in casual work, and therefore are more likely to lose work as employers respond to wider economic conditions [38]. We also found that workers in jobs with poor psychosocial job quality were more likely to fall out of the labour market. This makes sense, considering these jobs are characterised by high levels of job insecurity (as well as low control, high demands and pay inequity). Our results then suggest that workers perceptions that their jobs are tenuous are actually borne out in reality [40]. These findings highlight that people in certain jobs–particularly low status and lower quality work–are more likely to experience job loss. This is problematic in terms of health inequalities, considering research demonstrating the effects of unemployment on health [3].

### The effect of disability on exit from employment

It is worth noting that the presence of a disability influenced exit from employment above health, as measured by the SF-36. Considering this, what else about disability (other than health) could explain the greater exit from the labour market? We would speculate that these factors could include discrimination and bullying, as identified in past research [41]. There is increasing recognition of the mental and physical health impacts of being exposed to workplace bullying [42] (defined as a process in which an employee is subjected to frequent negative acts (e.g. at least once a week) for a relatively long period of time (e.g. 6 months) by peers or superiors, against which defence or retaliation is hindered by the recognition of a formal or informal power imbalance [43]). Research also suggests that greater exposure to workplace bullying is associated with higher sickness absence [44] and levels of job insecurity and intention to leave a job [45]. Hence, exposure to these negative experiences while in the workplace may contribute to exit from the labour market among workers with disability. Aside from explicit bullying, past research has identified lack of support from colleagues and supervisors as being a factor contributing to the wellbeing of workers with disabilities [46]. This support is being particularly necessary in easing workplace accommodations, which may also be associated with a considerable source of stress for workers with disabilities [46]. There is also a strong likelihood that other negative working conditions, such as high job demands and low job control, also contribute to the attrition of people with disabilities from the labour market. Indeed research suggests that people with disabilities are more likely to experience these stressors [8].

### Limitations

There has been a considerable amount of research demonstrating complexities in the measurement of disability [19, 47, 48], and we found this was also a challenge in the current study. The majority of our sample reported disability as a fluctuating condition. To take this into account, in addition to looking at individuals who report disability at all or no waves we conducted a detailed sensitivity analysis exploring the effects of altering disability measurement (e.g., between 1% to 24% of their contributed observations, 25% to 49% of their contributed observations, 50% to 74% of their contributed observations, and 75% and 100% of their contributed observations). The findings of these analysis were consistent with the main results reported in the main body of the paper, although lacked precision and were not statistically significant. Other limitations concern issues related to generalizability. A potential explanation for the differences in the study sample and the general population of people with disabilities is that we included only those persons who were in employment at the commencement of the study. Hence, the “healthy worker effect”, which sees the healthiest people being retained in work, may be having an effect here. Other issues include possible unmeasured confounding and dependent misclassification. In saying this, we would note that the outcomes of labour force status may be less prone to misreporting that more subjective factors, such as perceived psychosocial job quality. The strengths of this study are its large size and methodological approach, which allowed us to adjust for survivor bias introduced by censoring.

## Conclusion

In conclusion, it appears that workers vulnerable to dropping out of the labour market are lower skilled, precariously employed people, and those with disabilities. Employment-related predictors of exit from work among workers with disabilities did not differ from those workers without disabilities. In-depth research is needed to explore the specific employment-related experiences of persons with disabilities as we were only able to look at four psychosocial job stressors. There is also a need for more policy and research attention on this topic. Across OECD countries, disability employment policies have broadly focused disability discrimination legislation [49, 50], and increasing the funding of labour market programmes and vocational rehabilitation for people with disabilities [51]. However, there is little information on whether these are effective for workers with disabilities who exit of employment.

## Supporting information

S1 File Additional information on HILDA. Notes: HR = Hazard Ratio; L and U CI = Lower and upper confidence interval with 95% significance; p value = statistical significance at 95%. Notes: models also include the SF-36 (MCS and PCS), age, gender, education, household structure, and country of birth and household income.
(DOCX)

S1 Table Results of the Cox regression analysis, predictors of leaving employment, HILDA, 2001 to 2015 (persons = 14,565), effect modification by employment characteristics. Notes: HR = Hazard Ratio; L and U CI = Lower and upper confidence interval with 95% significance; p value = statistical significance at 95%. Models also adjust for the SF-36 (MCS and PCS), age, gender, education, household structure, region of residence, country of birth and household income.
(DOCX)

S2 Table Cox regression model, probability of leaving employment, by disability status, HILDA, 2001 to 2015. Notes: HR = Hazard Ratio; L and U CI = Lower and upper confidence interval with 95% significance; p value = statistical significance at 95%. Models also adjust for the SF-36 (MCS and PCS), age, gender, education, household structure, region of residence, country of birth and household income.
(DOCX)

S3 Table Cox regression model, probability of leaving employment, by proportion of time a person reported disability, HILDA, 2001 to 2015. Notes: HR = Hazard Ratio; 95% Lower CI = Lower confidence interval at 95% significance; 95% Upper CI = Upper confidence interval at 95% significance; p value = significance at 95% significance. Models also adjust for the SF-36 (MCS and PCS), age, gender, education, household structure, region of residence, country of birth and household income.
(DOCX)

S4 Table Cox regression model, probability of leaving employment, by unemployment and NILF, HILDA, 2001 to 2015. Notes: HR = Hazard Ratio; 95% Lower CI = Lower confidence interval at 95% significance; 95% Upper CI = Upper confidence interval at 95% significance; p value = significance at 95% significance. Models also adjust for the SF-36 (MCS and PCS), age, gender, education, household structure, region of residence, country of birth and household income.
(DOCX)
