# Supplementary material for: Employment predictors of exit from work among workers with disabilities: A survival analysis from the household income labour dynamics in Australia survey
Source: PLoS One. 2018 Dec 7;13(12):e0208334. doi: 10.1371/journal.pone.0208334 (PMC6285973; doi:10.1371/journal.pone.0208334)
Supplement: S1 File — Notes: HR = Hazard Ratio; L and U CI = Lower and upper confidence interval with 95% significance; p value = statistical significance at 95%. Notes: models also include the SF-36 (MCS and PCS), age, gender, education, household structure, and country of birth and household income. (DOCX) [file pone.0208334.s001.docx]

**S1 File. Additional information on HILDA**

The response rate to wave 1 was 66%. (Wilkins, 2013). The survey covers a range of dimensions including social, demographic, health and economic conditions using a combination of face-to-face interviews with trained interviewers and a self-completion questionnaire. Although data are collected on each member of participating households, interviews are only conducted with those older than 15 years of age.

The initial wave began with a large national probability sample of Australian households occupying private dwellings (Wilkins, 2013). Additional participants have been added to the sample as a result of changes in household composition. For example, if a household member left his or her original household (e.g. children left home, or a couple separated), then all new persons living with the original sample member are included. Inclusion of these new households is the main way in which the HILDA survey maintains sample representativeness.

**References**

Wilkins, R. (2013). *Families, Incomes and Jobs, Volume 8: A Statistical Report on Waves 1 to 10 of the Household, Income and Labour Dynamics in Australia Survey.* . Melbourne: Melbourne Institute of Applied Economic and Social Research Faculty of Business and Economics.
