# Supplementary material for: Employment predictors of exit from work among workers with disabilities: A survival analysis from the household income labour dynamics in Australia survey
Source: PLoS One. 2018 Dec 7;13(12):e0208334. doi: 10.1371/journal.pone.0208334 (PMC6285973; doi:10.1371/journal.pone.0208334)
Supplement: S1 Table — Notes: HR = Hazard Ratio; L and U CI = Lower and upper confidence interval with 95% significance; p value = statistical significance at 95%. Models also adjust for the SF-36 (MCS and PCS), age, gender, education, household structure, region of residence, country of birth and household income. (DOCX) [file pone.0208334.s002.docx]

S1 Table. Results of the Cox regression analysis, predictors of leaving employment,

HILDA, 2001 to 2015 (persons=14,565), effect modification by employment characteristics

|  |  | HR | L and U CI | p value |
| --- | --- | --- | --- | --- |
| Effect modification by occupational skill level | | | | |
| Occupation | High | 1 |  |  |
|  | Medium | 1.08 | 0.98, 1.25 | 0.195 |
|  | Low | 1.19 | 1.04,1.47 | 0.008 |
| Disability | No disability | 1 |  |  |
|  | Disability | 1.3 | 1.16, 1.47 | <0.001 |
| Effect | High*no disability | 1 |  |  |
| modification | Medium*disability | 0.94 | 0.81, 1.09 | 0.395 |
|  | Low*disability | 0.98 | 0.84, 1.14 | 0.791 |
| Effect modification by psychosocial job quality | | | | |
| Psychosocial job | High | 1 |  |  |
| quality | Low | 1.13 | 1.01, 1.26 | 0.029 |
| Disability | No disability | 1 |  |  |
|  | Disability | 1.31 | 1.15, 1.48 | <0.001 |
| Effect | High*no disability | 1 |  |  |
| modification | Low*disability | 0.96 | 0.83, 1.10 | 0.542 |
| Effect modification by employment arrangement | | | | |
| Employment | Permanent | 1 |  |  |
| arrangement | Casual or fixed-term | 1.71 | 1.55, 1.88 | <0.001 |
|  | Self-employed | 1.00 | .85, 1.18 | 0.991 |
| Disability | No disability | 1 |  |  |
|  | Disability | 1.37 | 1.25, 1.51 | <0.001 |
| Effect | Permanent*no disability | 1 |  |  |
| modification | Casual/fixed-term*disability | 0.85 | 0.75, 1.96 | 0.011 |
|  | Self-employed*disability | 0.92 | 0.75, 1.13 | 0.419 |

Notes: HR= Hazard Ratio; L and U CI=Lower and upper confidence interval with 95% significance; p value= statistical significance at 95%. Notes: models also include the SF-36 (MCS and PCS), age, gender, education, household structure, and country of birth and household income.
