# Supplementary material for: Employment predictors of exit from work among workers with disabilities: A survival analysis from the household income labour dynamics in Australia survey
Source: PLoS One. 2018 Dec 7;13(12):e0208334. doi: 10.1371/journal.pone.0208334 (PMC6285973; doi:10.1371/journal.pone.0208334)
Supplement: S2 Table — Notes: HR = Hazard Ratio; L and U CI = Lower and upper confidence interval with 95% significance; p value = statistical significance at 95%. Models also adjust for the SF-36 (MCS and PCS), age, gender, education, household structure, region of residence, country of birth and household income. (DOCX) [file pone.0208334.s003.docx]

S2 Table. Cox regression model, probability of leaving employment, by disability status, HILDA, 2001 to 2015.

|  |  | No disability (n=8623) | |  | Disability (n=5942) | |  |
| --- | --- | --- | --- | --- | --- | --- | --- |
|  |  | HR | L and U CI | p value | HR | L and U CI | p value |
| Occupation | High | 1 |  |  | 1 |  |  |
|  | Medium | 1.06 | 0.93 - 1.20 | 0.404 | 1.08 | 0.97 - 1.21 | 0.161 |
|  | Low | 1.13 | 0.97 - 1.31 | 0.105 | 1.21 | 1.07 - 1.37 | 0.002 |
| Psychosocial job | High | 1 |  |  | 1 |  |  |
| quality | Low | 1.15 | 1.03 - 1.28 | 0.011 | 1.06 | 0.97 - 1.17 | 0.183 |
| Employment | Permanent | 1 |  |  | 1 |  |  |
| arrangement | Casual or fixed-term | 1.68 | 1.52 - 1.86 | <0.001 | 1.47 | 1.34 - 1.60 | <0.001 |
|  | Self-employed | 0.97 | 0.82 - 1.15 | 0.760 | 0.95 | 0.83 - 1.08 | 0.414 |

Notes: HR= Hazard Ratio; L and U CI=Lower and upper confidence interval with 95% significance; p value= statistical significance at 95%. Models also adjust for the SF-36 (MCS and PCS), age, gender, education, household structure, region of residence, country of birth and household income.
