# Supplementary material for: Employment predictors of exit from work among workers with disabilities: A survival analysis from the household income labour dynamics in Australia survey
Source: PLoS One. 2018 Dec 7;13(12):e0208334. doi: 10.1371/journal.pone.0208334 (PMC6285973; doi:10.1371/journal.pone.0208334)
Supplement: S3 Table — Notes: HR = Hazard Ratio; 95% Lower CI = Lower confidence interval at 95% significance; 95% Upper CI = Upper confidence interval at 95% significance; p value = significance at 95% significance. Models also adjust for the SF-36 (MCS and PCS), age, gender, education, household structure, region of residence, country of birth and household income. (DOCX) [file pone.0208334.s004.docx]

S3 Table. Cox regression model, probability of leaving employment, by proportion of time a person reported disability, HILDA, 2001 to 2015.

|  |  | Disability reported between 1 and 24% of time | | | Disability reported between 25 and 49% of time | | |
| --- | --- | --- | --- | --- | --- | --- | --- |
|  |  | HR | L and U CI | p value | HR | L and U CI | p value |
| Occupation | High | 1 |  |  | 1 |  |  |
|  | Medium | 1.13 | 0.95 - 1.34 | 0.155 | 1.06 | 0.84 - 1.35 | 0.621 |
|  | Low | 1.26 | 1.05 - 1.52 | 0.014 | 1.13 | 0.88 - 1.46 | 0.335 |
| Psychosocial job | High | 1 |  |  | 1 |  |  |
| quality | Low | 1.10 | 0.95 - 1.26 | 0.190 | 1.10 | 0.92 - 1.33 | 0.295 |
| Employment | Permanent | 1 |  |  | 1 |  |  |
| arrangement | Casual or fixed-term | 1.52 | 1.33 - 1.73 | <0.001 | 1.43 | 1.19 - 1.71 | <0.001 |
|  | Self-employed | 0.94 | 0.76 - 1.17 | 0.597 | 1.04 | 0.80 - 1.35 | 0.764 |
|  |  | Disability reported between 50 and 74% of time | | | Disability reported between 75 and 100% of time | | |
|  |  | HR | L and U CI | p value | HR | L and U CI | p value |
| Occupation | High |  |  |  | 1 |  |  |
|  | Medium | 1.06 | 0.84 - 1.35 | 0.621 | 1.00 | 0.78 - 1.29 | 0.984 |
|  | Low | 1.13 | 0.88 - 1.46 | 0.335 | 1.07 | 0.81 - 1.41 | 0.644 |
| Psychosocial job | High |  |  |  | 1 |  |  |
| quality | Low | 1.10 | 0.92 - 1.33 | 0.295 | 1.01 | 0.82 - 1.25 | 0.924 |
| Employment | Permanent | 1 | 0.90 - 1.36 | 0.324 | 1 |  |  |
| arrangement | Casual or fixed-term | 1.43 | 1.19 - 1.71 | <0.001 | 1.39 | 1.13 - 1.72 | 0.002 |
|  | Self-employed | 1.04 | 0.80 - 1.35 | 0.764 | 0.85 | 0.63 - 1.14 | 0.276 |

Notes: HR= Hazard Ratio; L and U CI=Lower and upper confidence interval with 95% significance; p value= statistical significance at 95%. Models also adjust for the SF-36 (MCS and PCS), age, gender, education, household structure, region of residence, country of birth and household income.
