# Supplementary material for: Employment predictors of exit from work among workers with disabilities: A survival analysis from the household income labour dynamics in Australia survey
Source: PLoS One. 2018 Dec 7;13(12):e0208334. doi: 10.1371/journal.pone.0208334 (PMC6285973; doi:10.1371/journal.pone.0208334)
Supplement: S4 Table — Notes: HR = Hazard Ratio; 95% Lower CI = Lower confidence interval at 95% significance; 95% Upper CI = Upper confidence interval at 95% significance; p value = significance at 95% significance. Models also adjust for the SF-36 (MCS and PCS), age, gender, education, household structure, region of residence, country of birth and household income. (DOCX) [file pone.0208334.s005.docx]

S4 Table. Cox regression model, probability of leaving employment, by unemployment and NILF, HILDA, 2001 to 2015.

|  |  | Exit into Not in the Labour Force (NILF) | | | Exit into Unemployment | | |
| --- | --- | --- | --- | --- | --- | --- | --- |
|  |  | HR | L and U CI | p value | HR | L and U CI | p value |
| Disability | No disability | 1 |  |  |  |  |  |
|  | Disability | 1.25 | 1.16 - 1.36 | <0.001 | 1.40 | 1.21 - 1.62 | <0.001 |
| Occupation | High | 1 |  |  | 1 |  |  |
|  | Medium | 1.06 | 0.96 - 1.18 | 0.257 | 1.10 | 0.90 - 1.36 | 0.348 |
|  | Low | 1.12 | 1.00 - 1.25 | 0.060 | 1.42 | 1.13 - 1.77 | 0.002 |
| Psychosocial | High | 1 |  |  | 1 |  |  |
| job quality | Low | 1.00 | 0.92 - 1.09 | 0.951 | 1.61 | 1.33 - 1.94 | <0.001 |
| Employment | Permanent | 1 |  |  | 1 |  |  |
| arrangement | Casual or fixed-term | 1.55 | 1.43 - 1.68 | <0.001 | 1.95 | 1.67 - 2.26 | <0.001 |
|  | Self-employed | 1.05 | 0.93 - 1.18 | 0.459 | 0.59 | 0.44 - 0.80 | 0.001 |

Notes: HR= Hazard Ratio; 95% Lower CI = Lower confidence interval at 95% significance; 95% Upper CI= Upper confidence interval at 95% significance; p value= significance at 95% significance. Models also adjust for the SF-36 (MCS and PCS), age, gender, education, household structure, region of residence, country of birth and household income.
